# The Chicago Medical Register and Directory

**Published:** 1874-08-15

**Authors:** 


### THE CHICAGO MEDICAL REGISTER AND DIRECTORY.

SOME two years ago a first attempt was made to issue a Medical Register and Directory of Chicago and vicinity, similar in its scope to those issued by the profession in the larger Eastern cities.

The work was undertaken as a private enterprise by two or three of our well-known physicians. The gathering together of the material for the first issue of such a directory, necessarily involved the expenditure of a very considerable amount of time and labor on the part of the editors. In forming the list of physicians, who were in regular and good standing in the city, they called in the assistance of a committee consisting of representatives from each of the colleges and public institutions of the city. The list thus gathered together and passed upon was very good and reliable, as far as it went, although necessarily incomplete, on account of the difficulty of obtaining accurate information.

As a whole, however, the volume was carefully and conscientiously prepared, and met with the general approval of the profession.

The practical value and utility of such a register and directory has been so far demonstrated by this first attempt, as that a new and revised edition seems to be called for. The former editors being unwilling to take the responsibility of its re-issue, a meeting of the profession was called April 21st, 1874, to consider the best means of continuing the publication. This and a succeeding meeting April 28th, resulted in the organization of the Chicago Medico-Historical Society, which has undertaken to take charge of and continue the publication of the *Register and Directory*. To that end an editor was appointed with a committee on publication to assist him. A list of all persons styling themselves doctors or physicians was gathered together by the committee, and circulars sent out to each of them, asking for information regarding date and place of graduation, etc. Answers were received, however, from but a small proportion of those addressed.

The complete, entire list, together with such information as the committee was able to obtain regarding those named, either from the answers to circulars or otherwise, have now been submitted to the general Society at special meetings held during the past two weeks. Each name has been called up in succession and the vote passed, after brief discussion, as to whether it should appear in the *Register* or should be excluded. This, in many instances, has been an extremely difficult matter to decide upon. Persons who had returned no reply to the circular, and regarding whom no information could otherwise be obtained, were justly and necessarily excluded. In several more doubtful cases, however, names have been excluded on mere hearsay evidence or suspicion of irregular professional conduct, and this, when proper and satisfactory replies had been returned to the circulars. In justice to all parties more time and pains should have been taken to investigate the charges or obtain further information before passing the judgment of the Society.

Again we have to charge that the names of several as grossly irregular advertising quacks as exist in the city, have been passed for admission to the list, and that without the slightest pledge on their part of any desire or intention to reform. The mere fact that a man is not now, at the present time, distributing circulars or handbills or paying for puffs in the newspapers, has no bearing on the matter. What need is there to keep up this expense while reaping a rich harvest of business as a result of former exploits in that direction? Or, after scattering a few thousand puff advertisements, circulars, pamphlets, or books—it matters not which—broadcast through the city, and among our neighboring practitioners' patients, and families, who could not afford to repent and be generous—agree to burn up the few copies that might be left, to be good and do so no more—until next time?

The list, as now made up and passed upon by the Society for admission to the *Register*, is a simple farce. As a list of regular physicians in good standing in the city of Chicago, it is no more worthy of confidence than that given in the general city directory, and which includes everybody, male or female, who chooses to prefix the title of "doctor" to his or her name.

In order to be of any practical value whatever, the Register should have a full, complete, and carefully revised list of the regular physicians of our city. The formation of such a list must be the work of months, not of weeks. If the Society expect to have the support and endorsement of the general profession in this work, their hasty, inconsiderate action should be recalled, and the list again gone over. The unworthy names should be expunged and those omitted without sufficient reason be more carefully and deliberately considered. Take six months, if necessary, to do this work deliberately and carefully, rather than disgrace the Society and the profession of our city by the issuing of the list and directory in its present form. F. H. D.

